# The Current Situation and Future Prospects of Simulators in Dental Education

**DOI:** 10.2196/23635

**Published:** 2021-04-08

**Authors:** Yaning Li, Hongqiang Ye, Fan Ye, Yunsong Liu, Longwei Lv, Ping Zhang, Xiao Zhang, Yongsheng Zhou

**Affiliations:** 1 Department of Prosthodontics Peking University School and Hospital of Stomatology Beijing China; 2 National Clinical Research Center for Oral Diseases Peking University School and Hospital of Stomatology Beijing China; 3 National Engineering Laboratory for Digital and Material Technology of Stomatology Peking University School and Hospital of Stomatology Beijing China; 4 Beijing Key Laboratory of Digital Stomatology Peking University School and Hospital of Stomatology Beijing China; 5 NHC Key Laboratory of Digital Technology of Stomatology Peking University School and Hospital of Stomatology Beijing China; 6 The State Key Laboratory of Virtual Reality Technology and Systems School of Computer Science and Engineering Beihang University Beijing China

**Keywords:** dental simulator, dental education, virtual reality

## Abstract

The application of virtual reality has become increasingly extensive as this technology has developed. In dental education, virtual reality is mainly used to assist or replace traditional methods of teaching clinical skills in preclinical training for several subjects, such as endodontics, prosthodontics, periodontics, implantology, and dental surgery. The application of dental simulators in teaching can make up for the deficiency of traditional teaching methods and reduce the teaching burden, improving convenience for both teachers and students. However, because of the technology limitations of virtual reality and force feedback, dental simulators still have many hardware and software disadvantages that have prevented them from being an alternative to traditional dental simulators as a primary skill training method. In the future, when combined with big data, cloud computing, 5G, and deep learning technology, dental simulators will be able to give students individualized learning assistance, and their functions will be more diverse and suitable for preclinical training. The purpose of this review is to provide an overview of current dental simulators on related technologies, advantages and disadvantages, methods of evaluating effectiveness, and future directions for development.

## Introduction

Dental skills training is a very important part of preclinical learning in dental education and has a long history [1]. Pioneers of dental education began to use extracted teeth in dental skills education in the 1800s [2]. Later, Oswald Fergus invented the first phantom head simulator in 1894, which was used to teach oral anatomy and physiology to dental students [2]. Since then, the phantom head simulator has developed rapidly. Modern phantom head simulators include water spray, dental handpieces, and other necessary items [3], providing students with a more realistic environment for diagnosis and treatment. The dental simulator appeared in the 1990s [4,5] as a result of further research into methods of dental preclinical education, concern for patient safety, improvements in computer technology, and the inappropriateness of a clinical environment for the novice [1]. The arrival of the dental simulator marked a new era of dental preclinical education.

The dental simulator replicates both soft and hard oral tissues as well as providing a clinical diagnosis and treatment environment through virtual reality (VR). It also simulates the interaction force between the bur and the tooth, the mouth mirror, and soft and hard tissues through force feedback technology to reproduce the whole training process for dental clinical skills as closely as possible [6,7]. In recent years, the dental simulator has mainly been used for adult vocational training and university education [8].

Traditional preclinical dental skills training, which was based on a phantom head, extracted teeth, or plastic teeth [9,10], is generally used for practicing tooth preparation, for which the processes are irreversible. The acquisition of extracted teeth becomes more and more difficult, and the sensory feedback of preparing plastic teeth is different from that of real teeth [3,11]. The dental simulator, simulating realistic clinical conditions via VR and force feedback, makes training reversible, repeatable, and environmentally friendly [12,13]. Training via a dental simulator is varied [14] since different training content and tooth positions are available. These can be displayed in 3D on a computer screen for real-time evaluation by and feedback from teachers.

## Technologies Included in Dental Simulators

The dental simulator is a deep integration of computer and dental technology, mainly consisting of VR and force feedback technologies [15].

### Virtual Reality

**Figure 1:**
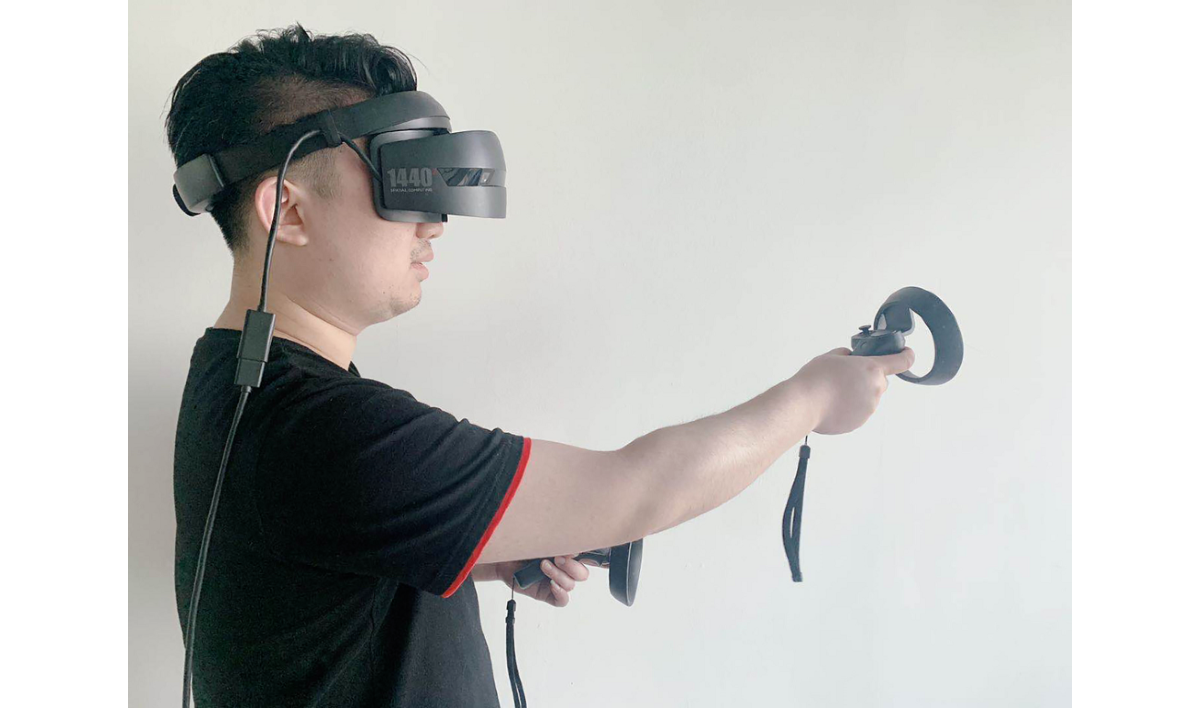
Head-mounted display and body motion sensors.

**Figure 2:**
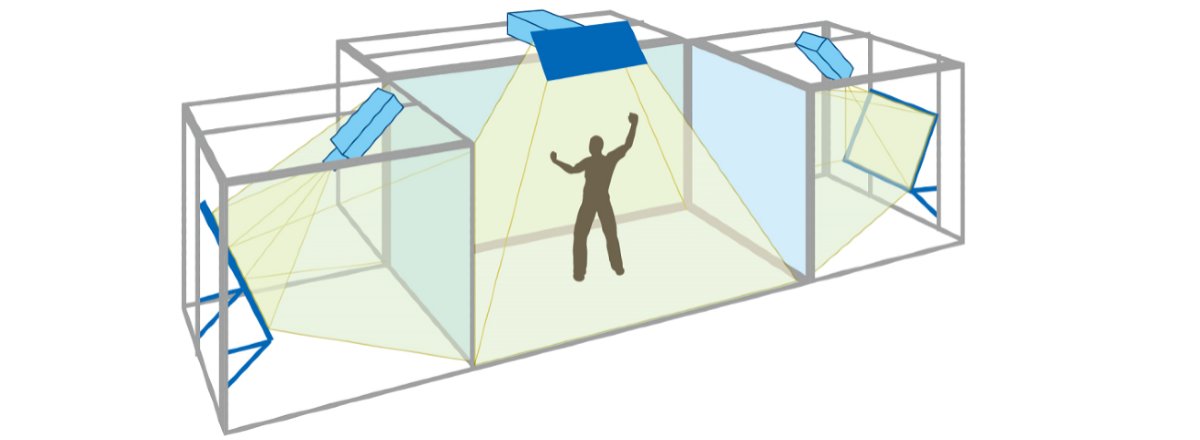
Cave Automatic Virtual Environment (CAVE).

VR uses computer technology [16] to generate a digital environment similar to the real environment in the visual, auditory, tactile, and other senses [17], which is used in many different fields. The operator interacts with and feels feedback from the virtual objects using specialized equipment [18]. A complete VR system consists of a stereo display device, a motion tracking device, an input device, and a computing platform. The stereo display device is usually a head-mounted display (HMD; Figure 1) [19]; another type of stereo display is the Cave Automatic Virtual Environment (CAVE; Figure 2) [20]. HMDs are more widely used than CAVEs because HMD application is more flexible and requires less space.

**Figure 3:**
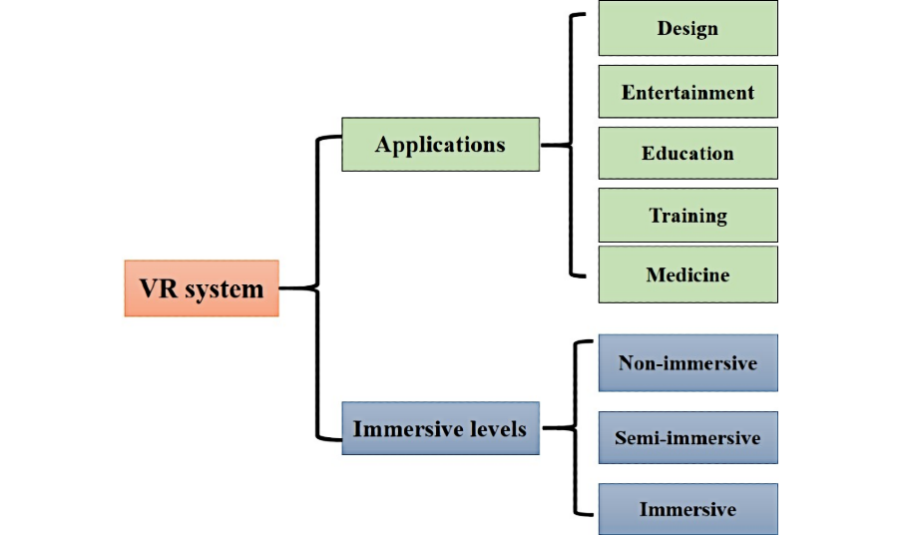
Applications and immersive levels of virtual reality (VR) systems.

VR systems are divided into three immersive levels based on the degree of stimulated senses and interactions: nonimmersive system, semi-immersion system, and immersion system (Figure 3) [21]. A nonimmersive system only reproduces images on desktops; an immersion system places the user in a complete virtual environment with the support of several sensory output devices including visual devices such as HMDs, audio devices, and haptic devices [22]; and a semi-immersion system provides the user with a simulated environment between the two above.

In the implementation of VR technology, the key points are modeling and interaction. In the medical field, images from computed tomography [7,23], magnetic resonance imaging (MRI) [24], and dental scanners [25] can be used to rehabilitate virtual models. Interactions mainly contain visual interaction and tactile sensation interaction, which are actualized by display device and force feedback device, respectively.

In the medical field, VR and related technology are gradually being applied in surgical training [26] and surgical navigation systems [27]. Combining VR with medical training is a new research field that has emerged with the development of computer science, sensor technology, and automation technology. A virtual operating environment with high fidelity and real-time performance can be created through virtual simulation technology [14]. The system is created and processed by a computer, with dedicated devices such as a helmet display and a force feedback handle that allow the user to observe and interact with the scene while experiencing multisensory feedback that give a near-realistic training effect [28].

### Force Feedback

Haptic devices can receive and transmit motion signals to improve the operator's sense of reality [29], which is important for dental training assisted by VR.

To simulate the tactile sense of real dental training as much as possible, the reaction force of the virtual object is calculated using the appropriate force generation algorithm in the VR system [30]. Due to the characteristics of human touch receptors, real-time haptic rendering requires a refresh frequency of at least 1 kHz [31]. For force feedback interaction in dental skills training, several kinds of interaction algorithms have been proposed for various scenarios, including teeth preparation, scaling, and bone drilling [32,33].

## Introduction to Existing Dental Simulators

### Overview

There is a wide variety of dental education simulators available, each with advantages and disadvantages in terms of training content, training process, hardware device, and software design. These are briefly described in Table 1.

**Table 1:** Comparison of dental simulators.

Characteristic			DentSim		Virtual Education System for Dentistry		IDEA^a^		Periosim		iDental		Simodont Dental Trainer		VirTeaSy		IDEAL^b^		Voxel-Man
**Hardware facility**																			
	Display type	2D display+ phantom head		2D display+ phantom head		2D display		3D glasses+ 2D display		2D display		3D glasses+ 2D display		3D glasses+ 2D display		2D display+ phantom head		3D glasses+ 2D display	
	Operation with two hands	Available		Available		Available		Available		Available		Available		No		Available		Available	
	Fixed finger rest	Yes		Yes		No		No		Yes		Yes		Yes		No		No	
	Ergonomic postures	Yes		Yes		No		No		Yes		Yes		Yes		Yes		Yes	
**Software design**																			
	Application field	Tooth preparation		Crown preparation		Manual dexterity training		Periodontal training		Periodontal training		Caries removal and crown preparation		Dental implant surgery		Dental radiography		Dental surgery	
	Fundamental skills	Yes		Yes		Yes		Yes		Yes		Yes		Yes		Yes		Yes	
	Clinical cases	No		No		No		No		Yes		Yes		Yes		Yes		No	
	Exam simulation	Yes		Yes		No		Yes		Yes		Yes		Yes		Yes		Yes	
	Repetitive practice	No		No		Yes		Yes		Yes		Yes		Yes		Yes		Yes	
	Practice at different levels	No		No		Yes		No		Yes		Yes		Yes		No		Yes	
	Individualized learning	No		No		No		No		No		No		Yes		No		No	
	Result evaluation	Yes		Yes		Yes		Yes		Yes		No		Yes		Yes		Yes	
	Force feedback	Yes		Yes		Yes		Yes		Yes		Yes		Yes		Yes		Yes	
VR^c^ immersive level			Non^d^		Non		Non		Non		Non		Non		Non		Non		Non

^a^IDEA: Individual Dental Education Assistant.

^b^IDEAL: Internet of Things–based dental education and learning.

^c^VR: virtual reality.

^d^Non: nonimmersive.

### DentSim

The DentSim system unit, consisting of a phantom head and dentoform, dental instruments, infrared sensors, infrared cameras, and two computers, was born in 1997 [34]. The infrared cameras can capture the orientation and movement of resin teeth and a handpiece so as to show the students’ work virtually on the computer screen in real time. The unit allows students to see the evaluation of their tooth preparation compared with the ideal preparation on the screen, while also providing them the ability to continue working on the resin teeth [35]. The training using DentSim is more efficient and standardized than that using traditional preclinical teaching methods [36]. The disadvantage of this unit is that it relies on physical resin teeth, which are disposable consumables.

### Virtual Education System for Dentistry

Virtual Education System for Dentistry is a dental simulator for prosthodontics developed by the Affiliated Stomatological Hospital of Nanjing Medical University and Suzhou Digital-health Care Company. The system contains the Virtual Learning Network Platform (VLNP) and Real-time Dental Training and Evaluation System (RDTES) [37]. Prior to practical work, students are requested to learn courses on the VLNP, including reading the operational instructions and predefined criteria of crown preparation and watching the standard operational videos. Afterwards, students can perform crown preparations on the phantom head under the guidance of the RDTES; the processes and results can be recorded by the RDTES. When the students finish their preparations, the RDTES can automatically assess the procedures and results of preparation based on the predefined tooth preparation criteria. As well, the students can visually compare their own procedures and results with the predefined assessment criteria on the computer screen [37,38].

### Individual Dental Education Assistant

Individual Dental Education Assistant (IDEA) is a VR hand flexibility training simulator consisting of a handheld stylus that simulates a dental handpiece and provides force feedback and a computer installed with simulation software. Unlike other dental simulators, IDEA is designed to enable students to be flexible and proficient in the use of dental handpieces by practicing removing predesigned virtual materials with different shapes (eg, straight line or circle). Therefore, IDEA aims to train dental students in hand flexibility, not to train students on a particular teaching component such as crown preparation or scaling. The main advantage of the system is its evaluation system. During the training process, two parameters determine the score obtained: drilling speed and drilling accuracy. Deviation from the trajectory or to an inappropriate depth can lead to a decrease in accuracy, and this is displayed as an accuracy bar on the screen. Complete depletion of the bar means that the student fails the test [39]. Some researchers have reported that IDEA could improve students' performance in the dental skill test; in addition, it can be used to identify students with troubles with hand flexibility at an early stage that may allow for early intervention to prevent failure [40,41].

### PerioSim

PerioSim, consisting of a stereoscopic display, a computer, and a haptic device, allows students to use virtual dental instruments to visualize and detect caries and periodontal diseases in a haptic environment [12]. The system is available online for students and allows teachers to upload different training programs, which can be downloaded and replayed by students at any time, making this system convenient and efficient [35]. Steinberg et al reported that the image display and force feedback were very realistic for teeth and dental instruments but not for gingival tissue [42].

### iDental

*i*Dental is a periodontal skill education simulator developed by Peking University School and Hospital of Stomatology and Beihang University, which can simulate periodontal examinations and treatment procedures including periodontal probing and calculus detection and removal. Unlike PerioSim, the device mainly uses a 2D monitor. However, it is equipped with an odontoscope handle, so it can be used to practice two-handed cooperative operation, making it realistic. *i*Dental also has a basic periodontal knowledge teaching module, which enables students to review basic knowledge before operation training. A combination of the two training parts improves the teaching effectiveness [43].

### Simodont Dental Trainer

Simodont Dental Trainer is a widely used teaching simulator for dental skills training that is currently available in many dental schools. It mainly includes modules for hand flexibility, cariology, crown and bridge preparations, clinical cases, and a full mouth simulation experience [13,44]. One of the highlights of the system is that an X-ray of the working tooth is attached to each individual case, which can allow students to make a diagnosis assisted by both the appearance of teeth and the X-ray films [13].

The system does have some disadvantages. It requires 3D glasses for 3D display. Excessive training on a single tooth fails to create a realistic sense of manipulation. The training process for crown preparation is single-jawed and does not fully mimic the narrow operating space of the mouth. Besides, it cannot be used to train students about positioning requirements because operation postures during training are fixed and visual angle conversion requires manual rotation of the rotary button.

### VirTeaSy Project

The VirTeaSy project is a dental implant training simulator and is composed of VirTeaSy Scan Implant and VirTeaSy Implant Pro. VirTeaSy Scan Implant is used for implanting scheme designs by students. Radiographs are used by students to perform the implant treatment plan, including the implant’s characteristics (shape, diameter, length) and its location (location, angle, insertion depth) in the jaw. When the treatment plan is complete, it can be compared with the design planned by an expert that is stored in the database to identify where improvements could be made in their own plans. In addition, the device's database contains cases of varying difficulty, so students with different skill levels can use it to practice accordingly [45].

VirTeaSy Implant Pro is a virtual implant surgery training system, which allows students to perform surgery cases planned in VirTeaSy Scan Implant. VirTeaSy Implant Pro is supplemented by an auxiliary system that can alert students if the drilling’s location, angle, and depth are incorrect as well as if there is overheating of the bone. In addition, the VirTeaSy Implant Pro has a display through which the teacher can interact with the student in real time and assist the student as necessary. VirTeaSy Implant Pro can improve students’ skills in bone mineral density perception through force feedback, thus allowing them to perceive whether their bone density measurements in VirTeaSy Scan Implant match the reality [46]. VirTeaSy Scan Implant and Implant Pro complement each other, forming an efficient learning tool [45].

### Internet of Things–Based Education and Learning System

The Internet of Things–based education and learning (IDEAL) system is an oral radiology education simulator. The simulator is mainly used for teaching students to take intraoral X-ray images without using X-rays. The IDEAL system consists of a simulator cone, simulator main body, sensor, detector, and stool. Training contents comprise basic education before X-ray imaging, information on X-ray imaging techniques (such as periapical radiography, bisecting angle technique, and paralleling technique), a test bank, and an evaluation and feedback system. During the training process, students can practice taking X-ray images for different tooth positions by adjusting the angle of the X-ray tube to improve their skills in taking X-ray images. At the end of training, students submit their own imaging results to the system and receive automatic feedback and evaluations from the system. The system allows students to avoid using radiographic devices while learning to take dental films, reducing the risk of radiation exposure to both students and patients. The system is safe and affordable [47].

### Voxel-Man Simulator

The Voxel-Man simulator is a VR surgical simulator that primarily consists of a 2D monitor, simulated surgical operating handle, foot pedal, and 3D glasses. At present, the device is mainly used to simulate apical surgery, such as apical resection and apical cystectomy [48].

The system provides different training modes depending on the trainees’ level, with different display interfaces and different operating instructions. The three modes to choose from are primary mode, advanced mode, and exam mode. In primary mode, the lesions and surrounding important anatomical structures (such as the alveolar nerve) are marked with bright colors. This not only helps operators to understand the anatomical characteristics of the corresponding surgical area, but also reminds them of the scope and amplitude of the operation at all times to help them avoid damaging these important anatomical structures. When the surgical instrument is close to an important anatomical structure, the system will emit a danger alarm. In advanced mode and exam mode, some of the functions and hints are turned off [48,49].

In addition, the system can record the operation process for later replay so that the operator can identify errors and mistakes to improve on [48]. The operator can stop the operation at any time and can undo a wrong step or restart a new one, saving a lot of time. Cases vary in difficulty, allowing operators with different experience levels to practice.

## Advantages of Dental Simulators

Compared to phantom-based traditional training methods, dental simulators have many strengths that will offer students a better learning environment. Besides dental operation skills, students are also able to acquire relevant theoretical knowledge through dental simulators [37]. Since the dental simulators allow repeatable and reversible preclinical training of clinical skills [50,51], they give students a more flexible training experience [14]. They also allow digital objective evaluation and tutorial feedback [34,52] by recording the training processes [53]. In addition, training in dental simulators is more clinically relevant [54] because they recreate situations that are similar to those encountered in a real clinical environment [14]. It is certain that dental simulators can eliminate the risk of treatment and enhance the safety of patients [55,56]. Previous studies showed that training using dental simulators can save the time of faculty [50,57] and allow students to practice repeatedly whenever they want until they achieve mastery. Some studies reported that training in dental simulators can reduce training time compared with traditional training methods [57]. Therefore, the application of dental simulators in teaching can make up for the deficiency of traditional teaching methods and reduce the teaching burden, improving convenience for both teachers and students.

## Current Disadvantages of Dental Simulators

The dental education simulators that have been described all have certain disadvantages in terms of both hardware and software.

### Disadvantages of Hardware

#### The Stereo Vision of the Display Is Not Visible Enough and the Resolution Is Not High Enough

Currently, 3D displays in dental simulators are relatively behindhand. To achieve 3D display, most simulators use 3D glasses; these can slightly change the color of the oral tissues and can produce unpleasant effects such as vertigo and nausea [45,58]. In addition, 3D glasses change the image quality to a certain extent, reducing the resolution of the image. The display of oral tissue needs to be precise since the dental operation needs to identify subtle differences between different kinds of tissues, so a lower resolution may result in inaccuracy in the operation [59]. Image display can also affect the VR immersiveness, so it is necessary for researchers to find a higher resolution of 3D display to replace 3D glasses.

#### No Fixed Physical Finger Rest

A stable finger rest is of great significance in dental operations because of the small intraoral space and the requirement for precise dental operations [15,43]; without it, accidental injury to the surrounding soft and hard tissues may occur. Training on the use of finger rests is crucial in dental skills training, so finger rests should be provided for optimal simulation during dental skill training.

#### Lack of Bimanual Cooperative Operation

Bimanual operation is extremely important in dental operations. In general, the left hand of the operator manipulates an odontoscope to stretch and protect the soft tissues and reflect light, to ensure accurate and safe intraoral manipulation of the instrument held in the right hand. For optimal simulation of an operation on a real patient, training on bimanual operation in a dental simulator is essential [53,60].

### Disadvantages of Software Design

#### Simulation of Force Feedback Is Not Realistic Enough

In clinical operations, dentists continually judge the process to determine whether to continue the operation by perceiving the different force feedback of the different oral tissues. Therefore, the fidelity of force feedback in the simulator is vital to dental training [27]. At present, the force feedback of oral simulators is based on a force-generating algorithm. This is different from the force feedback of a real instrument in contact with the oral tissue, especially soft tissue. Consequently, researchers need to work on improving the fidelity of force feedback in the future [14,61].

#### Simulation of Soft Tissue Deformation Is Not Realistic Enough

An oral environment simulation includes simulation of tongue and facial tissue deformation [62]. When a simulated surgical instrument collides with the deformable object, the object needs to deform accordingly. Deformation simulation is based on the physical properties of the soft body, such as density and elasticity. To express these properties, a physical model must be established; these commonly include the mass-spring method [63,64] and the finite element method [65]. The mass-spring method is simple and commonly used, but it is hard to maintain tissue volume information and set the stiffness of the spring. The drawback of the finite element method is that it is hard to use in real-time simulation [65]. Therefore, a physical model with higher calculation efficiency and more accurate simulation that can better represent the physical characteristics of oral tissues needs to be established.

#### The Training Content Is Insufficient

At present, many dental simulators do not have different degrees of difficulty in training, so students cannot learn step by step in the process of training. Moreover, the simulators have only basic skills training and lack the application of skills in clinical cases, so they cannot assess students' progressive mastery of skills. More comprehensive and systematic training content should be developed in the future [15].

#### The Evaluation of Training Results Cannot Be Accurately Quantified

It has been reported that professional instruction and performance feedback are beneficial for students’ skills acquisition [66,67]. Some simulators cannot give an accurate quantitative evaluation of the student's operational results after training, so students do not know whether their operation results meet the requirement or not.

**Figure 4:**
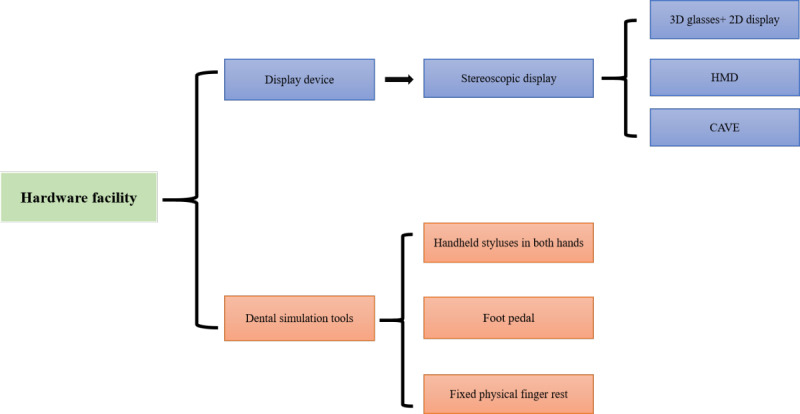
Hardware facilities required for a dental simulator. CAVE: Cave Automatic Virtual Environment; HMD: head-mounted display.

**Figure 5:**
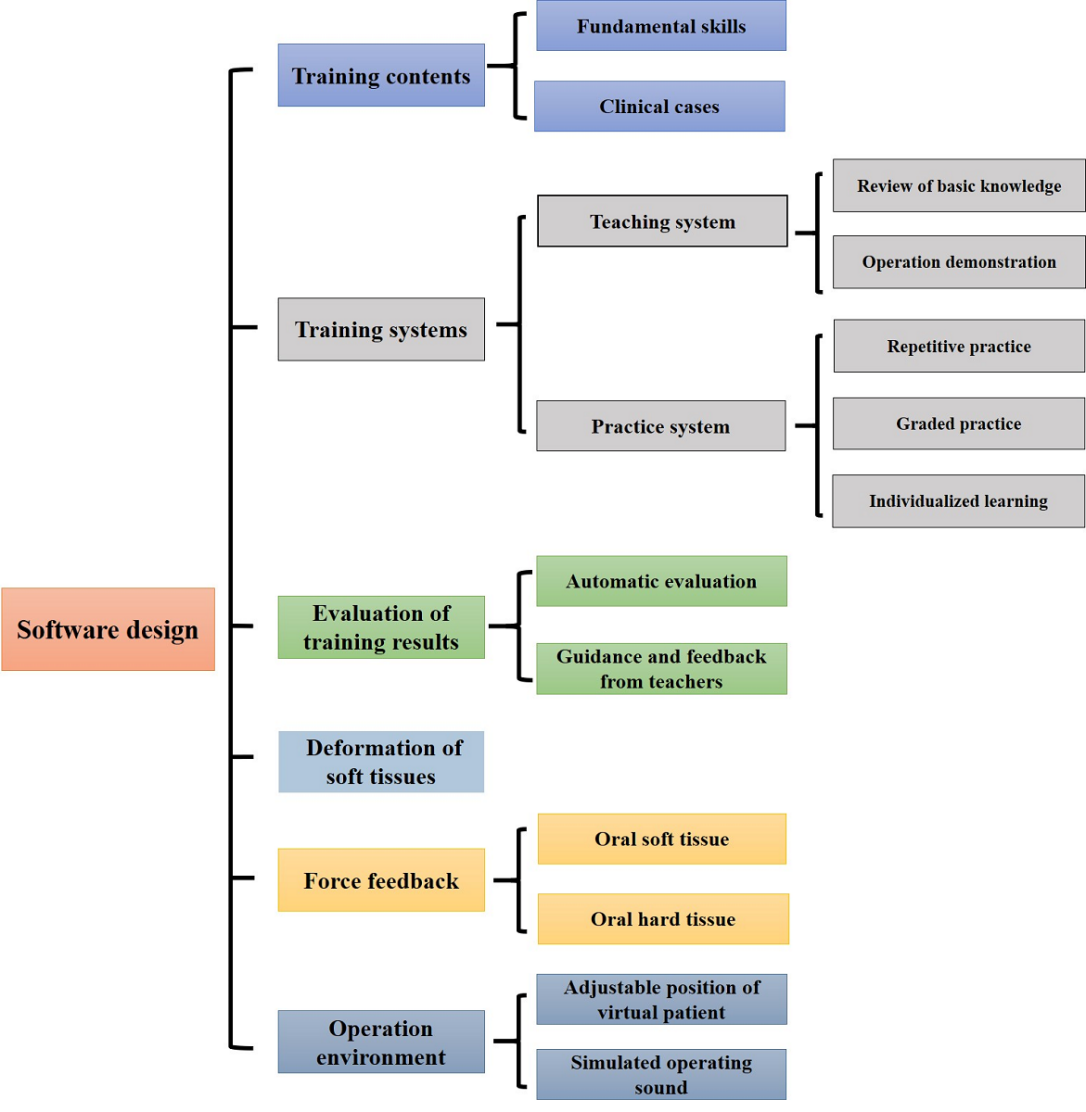
Software design required for an ideal dental simulator.

In summary, a good dental skills training simulator should be able to overcome these shortcomings, and its comprehensive features are summarized in Figures 4 and 5.

## Effectiveness Evaluation of Dental Simulators

### Methods for Effectiveness Evaluation

The effectiveness of VR systems can be evaluated using two analytical methods, qualitative and quantitative [15,43], which can be analyzed by questionnaires and comparative studies, respectively.

#### Questionnaires

Questionnaires are mainly used to investigate the subjective experience of people using the equipment, providing possible ways to improve the next iteration of the simulator. The content of the questionnaire is mainly based on the relationship between the user and the simulator. The simulator has two categories of users: trainers and trainees. For trainers, the device is a teaching tool and therefore needs to be evaluated for its auxiliary role in teaching, such as whether it reduces teaching cost or improves teaching efficiency. For trainees, the device is a learning tool and needs to be evaluated for learning effectiveness and construction of a learning environment [68]. Therefore, the content of the questionnaire should be designed according to the characteristics of the respondents.

The content of the questionnaire is mainly classified into two categories. The first category is the evaluation of the software and hardware of the device, involving force feedback, 3D modeling, the ease and convenience of using the device, the simulation degree, and the design of the device. Wang et al proposed a qualitative evaluation architecture based on the analysis of function components, which included the performance and usability of the simulator [15]. The second category is the evaluation of the teaching effect of the device, which involves the subjective evaluation of the equipment effect by users [69]. The main questions in such questionnaires are whether the students think that the simulator assisted their study, improving their understanding of the related curriculum, and whether they are willing to use the simulator in their future study [70]. In addition, Venkatesh et al made the Unified Theory of Acceptance and Use of Technology questionnaire proposal to measure the acceptance of a new technology [71], and Bravo et al applied it to the use of dental simulators and concluded that it can assess the acceptance of dental simulators in dental education [72].

#### Comparative Studies

Comparative studies are mainly suitable for quantitative evaluation of objective function of a simulator, such as evaluating the role of the simulator in education compared with traditional teaching methods [73,74], the accuracy of the simulator at evaluating different kinds of trainees [46,75], and its accuracy in predicting the skill levels of students [76]. Barsom et al proposed a matrix of validity type to train medical professionals and classified the existing research methods including surface validity, content validity, construct validity, concurrent validity, and predictive validity [77,78].

Construct validity is the most widely used criterion in the functional evaluation of simulators [79]. The objects of the study of construct validity are populations with different clinical experience and skills in a corresponding specialty, such as experts and students [80]. By comparing the relevant parameters of using the simulator in different groups, construct validity of the simulator aims to distinguish different levels of dental skills. Dental trainees with diverse training time in a study by Mirghani et al [75] and dental students in their first year and experienced dentists in a study by Eve et al [45] were chosen to be trained using the simulator to evaluate its construct validity.

### Influence Factors

Dental skills training takes the form of gradual acquisition, so the proficiency of training using the simulator can improve along with increasing familiarity with the training content. Urbankova et al concluded that repeating the training led to learning the test content, which could affect the results of the study [40]. Since VR is a new technology, teachers’ sensitivity to the simulator may depend on their age, which could affect the results of the experiment. Therefore, questionnaires are often designed to eliminate the influence of these two factors [81].

Training and demonstration before the experiment, which allow objects to know the experimental flow and operational specification and eliminate the interference factors, are necessary [47]. Seymour et al tested trainees' visual ability, perception ability, and mental condition to exclude interference factors that affected experimental results [82].

### Effectiveness of Dental Simulators

To date, many pilot tests have been conducted to evaluate the validity of dental simulators in endodontics [3,83], periodontics [12,42,43,84,85], oral and maxillofacial surgery [65,86-88], dental radiography [41], prosthodontics [37,59,85], implantology [89,90], and orthodontics [90]; the specific applications of dental simulators in dental education are summarized in Table 2.

**Table 2:** Applications of dental simulators in dental fields.

Dental fields	Training contents
Endodontics	Dental caries detection Caries removal Light-curing skills Endodontic cavity preparation
Periodontics	Periodontal probing Calculus detection and removal Ultrasonic scaling
Oral and maxillofacial surgery	Dental anesthesia training Maxillofacial palpation Dental extraction skills Orthognathic surgery Dental surgery
Dental radiography	Intraoral X-ray imaging
Prosthodontics	Tooth preparation
Implantology	Implanting scheme design Implant drilling skills
Orthodontics	Training and treatment planning in orthodontics

Many researchers are positive about the roles that dental simulators play in skills training. Al-Saud et al reported that students could gain basic manual dexterity at a quicker pace when they practiced skills with the guidance of experienced faculty, and training with dental simulators helped students to retain the skills they had learned [91]. A study performed by Plessas showed that students could develop a better understanding of the material by using dental simulators since they create a more varied learning environment compared with traditional training methods [74]. Suebnukarn et al evaluated the effectiveness of dental simulators in cavity preparation and concluded that haptic VR simulators are equivalent to conventional dental phantom heads in reducing operation errors [92]. Other research provided evidence that students can improve their tooth extraction skills [93], dental radiology skills [47], and implant skills [32,90] through dental simulators. Dental simulators were also reported to be able to enhance students’ confidence [94] and improve their attitudes toward patients [53] and abilities to discern and solve medical emergencies [70,95,96]. Some studies focused on students’ acceptance of dental simulators and found that most students are willing to learn with dental simulators, which would boost their enthusiasm to learn [97]. Therefore, many research studies indicated that dental simulators had the potential to be an alternative to conventional training methods [36,52,98].

However, as mentioned above, there are still some disadvantages of dental simulators that can’t be ignored, and these disadvantages may directly influence the effectiveness of dental simulators. In addition, the effectiveness of some dental simulators has not been validated [88,98]. Therefore, it is suggested that dental simulators cannot completely replace traditional skill training methods. The automated evaluation and tutorial feedback offered by dental simulators are considered to be complementary components to traditional methods [84]. Some studies concluded that a combination of traditional and virtual methods would be an optimal approach to choose in skills training [52,99,100].

## Prospects of Dental Education Simulators

Current simulators are deficient in stereovision, video resolution, force feedback, instructional content, and outcome assessment; in response to these issues, we offer an outlook for their future development.

### Visualized Analyses of Education Data Based on Big Data Technology

By applying big data technology to medical education, we can establish statistical models by the visual analysis method [101] via educational data mining and learning analysis [102], which allow trainers to analyze and understand the learning status of trainees intuitively. Currently, big data technology has been applied in educational analysis of massive open online courses [103] and learning effectiveness prediction for medical courses [101].

A similar big data analysis platform can be set up in dental skills training. We can collect dental operation data and establish a database of correctly performed operations. Then, a scientific evaluation system to score the training results by comparing them with the correctly performed operations can be created. Trainers can identify information on general problems in operations, as well as problems experienced by the individual trainee, to analyze and predict trainees' ability to master the skills, error-prone points, and the pass rate of examinations. Based on the analysis, trainers can create or adjust different plans for all trainees to enhance their learning experience and performance. Therefore, the combination of big data and education will make it possible to deeply understand and study the training process in order to provide more trainees with quality training.

### Video Transmission With Quicker Speeds and Less Delay Supported by 5G

The development of 5G technology will allow transmission links with high bandwidth and low delay [104-106]. In the virtual oral training system, calculations are often performed on the server side to improve accuracy. The use of 5G technology will greatly increase the transmission rate of high-definition video data from the server host to the display device, reducing the response delay [107-109]. This will provide users with more reliable visual and tactile feedback and improve comfort [110].

Currently, the 5G quality of experience framework is proposed to solve the problem of transmitting ultra-high-definition video on the 5G network [111]. This could provide a basis for the establishment of a VR dental educational video network. Large-scale synchronous training and one-to-one demonstrations could be performed via 5G transmission to improve training efficiency.

### Improvement of the Simulation of Force Feedback by Deep Learning

Deep learning techniques are widely used to find rules from a large amount of data, abstract problems through neural networks, and establish input and output mapping.

In the field of haptics, deep learning has been used to obtain haptic properties of objects from their images [112], whereby force feedback devices can directly generate a sense of force feedback that mimics the real physical properties without having to manually adjust physical parameters.

We can use deep learning to obtain the force feedback properties of teeth and build different deep learning models for different parts of the teeth. Therefore, the instrument would have different force feedback in contact with normal teeth, dental caries, tooth enamel, dentin, and other areas.

Deep learning can also play a role in oral modeling. Now, deep learning has been widely used in medical image analysis and modeling [113]. Through this type of method, we can easily extract relevant regions from computed tomography or MRI data and reconstruct mesh data [114]. These technologies can be transferred to oral cavity modeling, which can greatly facilitate model construction in a virtual surgery system and quickly establish a personalized oral cavity model.

### Improvement of the Immersiveness of Dental Simulators by Augmented Reality

Augmented reality technology is an extension of VR technology, combining virtual images with real environments to support interaction with virtual objects in real time.

Nowadays, augmented reality technology has begun to be used in the medical field. Based on an augmented reality helmet (HoloLens by Microsoft), various applications have been developed such as surgical navigation applications [115] and intelligent medical management systems [116]. HoloLens also supports a display of high-definition video derived from high-performance computing.

The virtual tooth and the phantom head can be combined with augmented reality technology in a dental training system. Students can feel the phantom head directly with their hands and interact with a virtual tooth through a force feedback device. At the same time, high-definition images allow users to observe finer tooth details, which can greatly improve the reality and the immersiveness of the experience. The users can also observe patients from multiple views, which will greatly enhance the trainees' understanding and learning of training objectives [117,118]. Therefore, augmented reality will have a significant effect on dental education in the future.

## Strengths and Limitations

The strengths of this paper are as follows: First, we have come up with a summary of comprehensive features that a good dental simulator should possess based on the analysis of the advantages and disadvantages of current dental simulators. Second, this paper proposes the future prospects of dental simulators and provides researchers with new ideas for further studies.

Our paper also has some limitations. First, our paper lists the nine dental simulators that are commonly mentioned and studied in most articles instead of all kinds of dental simulators, which may have a little effect on the results of our study. Second, our study is focused on VR- and haptic-based dental simulators; as a consequence, some relevant papers may not be included due to inappropriate use of keywords.

## Conclusion

Despite the fact that dental simulators are not currently able to rival traditional training modalities for skills training in some disciplines, they still have some advantages over traditional methods, and their effectiveness has been validated in some cases. More studies should be conducted to improve the force feedback, video transmission, and immersiveness of dental simulators. With scientific and technological development, dental simulators that gradually combine with big data, cloud computing, 5G, and deep learning technology will offer students individualized learning assistance, and their functions will be more diverse and suitable for preclinical training.

## Abbreviations

CAVE: Cave Automatic Virtual Environment
HMD: head-mounted display
IDEA: Individual Dental Education Assistant
IDEAL: Internet of Things–based education and learning
MRI: magnetic resonance imaging
RDTES: Real-time Dental Training and Evaluation System
VLNP: Virtual Learning Network Platform
VR: virtual reality
